# RLN2 Is a Positive Regulator of AKT-2-Induced Gene Expression Required for Osteosarcoma Cells Invasion and Chemoresistance

**DOI:** 10.1155/2015/147468

**Published:** 2015-07-01

**Authors:** Jinfeng Ma, Hai Huang, Zenggang Han, Changzheng Zhu, Bin Yue

**Affiliations:** ^1^Department of Spine, The Affiliated Hospital of Qingdao University, Qingdao, China; ^2^Department of Orthopedics, Linyi People's Hospital, Linyi, Shandong, China; ^3^Department of Dialysis Room, Zhangqiu Hospital of Traditional Chinese Medicine, Zhangqiu, Jinan, China; ^4^Department of Neurology, People's Hospital of Zhangqiu, Jinan, China

## Abstract

The aim of the study was to determine the effect of H2 relaxin (RLN2) on invasion, migration, and chemosensitivity to cisplatin in human osteosarcoma U2-OS and MG-63 cells and then to investigate the effect of RLN2 on the AKT/NF-*κ*B signaling pathway. The expression of RLN2, p-AKT (Ser473), and p-ERK1/2 (Phospho-Thr202/Tyr204) proteins was detected by western blot in OS tissues from 21 patients with pulmonary metastatic disease, and the correlation between RLN2 and p-AKT or RLN2 and p-ERK1/2 expression was investigated. RLN2 expression was inhibited by RLN2 siRNA transfection in the MG-63 cells. RLN2 was overexpressed in the U2-OS cells by treatment with recombinant relaxin. The results showed that positive relation was found between RLN2 and p-AKT expression in tissues of OS. Silencing RLN2 inhibited cell migratory and invasive ability and angiogenesis formation and increased the chemosensitivity to cisplatin in MG-63 cells. RLN2 overexpression promoted migratory and invasive ability and angiogenesis and increased the chemoresistance to cisplatin in U2-OS cells. Silencing RLN2 inhibited the activity of AKT/NF-*κ*B signaling pathway in MG-63 cells, and vice versa. Blockage of both pathways by specific inhibitors abrogated RLN2-induced survival and invasion of OS cells, and vice versa. Our results indicated RLN2 confers to migratory and invasive ability, angiogenesis, and chemoresistance to cisplatin via modulating the AKT/NF-*κ*B signaling pathway in vitro.

## 1. Introduction

Osteosarcoma (OS) remains the most common primary malignant bone cancer affecting children and adolescents [1]. Although the combination of modern surgery and systemic chemotherapy has improved OS treatment dramatically, no substantial change in survival has been seen over the past 20 years [2]. For this reason, understanding the mechanisms underlying OS as well as identifications of new molecular targets is of great importance.

H2 relaxin (RLN2) is a peptide hormone that is a member of the insulin-like superfamily. Relaxin-2 binds specifically to the LGR7 receptors also known as relaxin family peptide receptor 1 (RXFP1). The same applies to the LGR8 receptor that is currently called RXFP2. Signaling by relaxin-2 through its target receptors enhances the growth of pubic ligaments and ripening of the cervix during birth. Several groups, including ours, have demonstrated that H2 relaxin plays a role in OS carcinogenesis [3]. However, the mechanism that RLN2 functions in detail is not clear.

Members of AKT kinase family are central modulators in numerous signaling cascades, which regulate cell proliferation, survival, and progression of osteosarcoma [4–7]. Canonical NF-*κ*B activation was recently implicated in epithelial-mesenchymal transition (EMT), a change thought to herald tissue invasion and prophesize metastatic potential [8]. Activation of a so-called mesenchymal program (involving genes such as MMP-2/9 and alphavbeta3 integrins) was found to be dependent on NF-*κ*B activation in OS model, and reversal of EMT was triggered by NF-*κ*B inhibition [9–11]. Recently, many studies have found that NF-*κ*B activation was dependent on AKT phosphorylation (p-AKT) [12–17]. Jeong et al. have confirmed that RLN2-stimulated MMP-9 expression is dependent on NF-*κ*B activation in THP-1 cells [18]. Mirmohammadsadegh et al. have found that NF-*κ*B could upregulate the expression of vascular endothelial growth factor (VEGF) and intercellular adhesion molecule 1 (ICAM-1) in OS cells [19]. Another study has found relaxin could activate AKT/NF-*κ*B signal [20] and VEGF expression in wound macrophages [21]. We therefore suggest that RLN2 could regulate osteosarcoma invasion, which might be through AKT/NF-*κ*B (MMPs and VEGF) signal pathway.

Studies have found activation of AKT and extracellular signal-regulated kinase (ERK)1/2 increased the chemoresistance in many tumour cells, including human osteosarcoma cells [18–24]. Inhibition of AKT or ERK1/2 activation sensitized cancer cells to cytotoxic drug and that combination treatment with cytotoxic drug and AKT or ERK1/2 inhibitor resulted in greatly enhanced apoptosis and growth inhibition in cancer cells compared to treatment with either agent [18–24]. In pancreatic cancer, downstream targets of AKT sensitized cells to the apoptotic effect of chemotherapy by NF-*κ*B/Bcl-2 signalling pathway [25]. It has also been found that AKT2 inhibition sensitized gemcitabine-induced apoptosis in part via inhibition of NF-*κ*B activity in some cells [26]. In OS cells, genistein reversed the cancer's resistance to gemcitabine through abrogating the AKT/NF-*κ*B pathway [27]. It has recently been found that chemically synthesized AT-001, an analog of human RLN2, could suppress PC3 xenograft growth and sensitize PC3 xenografts to docetaxel, standard first-line chemotherapy for HRPC [28]. We have previously found the levels of RLN2 mRNA expression in OS tissue samples were significantly higher than those in the corresponding nontumor tissue samples [3]. Whether RLN2 could be important therapeutic targets for overcoming chemoresistance or whether RLN2 overcomes chemoresistance by AKT/NF-*κ*B pathway is not very clear to date. Previous study has found that RLN2 treatment of human endometrial stromal cells resulted in rapid activation of MAPK (or ERK) kinase (MEK) in THP-1 monocytic cells and in human smooth muscle cells, but not triggering the PI 3-kinase/AKT pathway, perhaps accounting in part for relaxin's unique biological profile [29]. In OS cells, whether MAPK (or ERK) kinase (MEK) was regulated by RLN2 or whether RLN2 overcomes chemoresistance by ERK1/2 pathway is not very clear. We tested the hypothesis that RLN2 signaling pathways could be an important therapeutic target for overcoming chemoresistance in OS cells, and RLN2 confers to OS chemoresistance in part by AKT/NF-*κ*B and/or ERK1/2 pathway.

In the present study, we provide evidence to support the hypothesis that RLN2 plays a role in invasiveness and chemosensitivity to cisplatin of human OS through AKT/NF-*κ*B pathway and/or ERK1/2 pathway. We conclude that RLN2 would be a good molecular target for OS therapy.

## 2. Materials and Methods

### 2.1. Patients and Tissue Samples

A total of 21 samples of OS tissues were obtained from patients with pulmonary metastatic disease who underwent surgery in our hospital (Department of Spine, the Affiliated Hospital of Qingdao University) from January 12, 2008, to October 26, 2011. The OS survey was performed with plain films and chest CT scans at first diagnosis. All the patients have no history of prior therapies with anticancer drugs or radiotherapy. Samples were snap-frozen in liquid nitrogen and stored at −80°C after they were resected. In all cases, informed consent was taken from related departments and persons, and the study had the approval from the Institute Ethics Committee of the Affiliated Hospital of Qingdao University.

### 2.2. Reagents

Cisplatin was purchased from Sigma Company (USA). Antibodies to RLN2, LGR7, p-Akt (Ser473), p-ERK1/2 (Phospho-Thr202/Tyr204), AKT, ERK1/2, NF-*κ*B (p65), MMP-9, VEGF, Bcl-2, GAPDH, RLN2 siRNA (h), and recombinant human RLN2 (B-29/A-24) were from Santa Cruz Biotech Inc. (USA). Constitutively active myristoylated AKT cDNA (myr-AKT) was from Upstate, Charlottesville, VA, USA. LY294002 was obtained from New England BioLabs (Beverly, MA, USA) and is a well-established inhibitor of PI3K-mediated activation of AKT. I kappa B alpha mutant (I*κ*B*α*M), a dominant negative NF-*κ*B (super repressor of NF-*κ*B activity), was from Santa Cruz Biotechnology Inc. (Santa Cruz, CA, USA). NF-*κ*B inhibitor BAY 11-7082 was from Shanghai, China.

### 2.3. Cell Culture

The human osteosarcoma cell lines MG-63 and U-2OS were obtained from the ATCC (Rockville, MD) and conserved in the central laboratory. They were incubated in RPMI 1640 medium containing 10% fetal calf serum (FCS, Gibco) and 1% antibiotics (P/S, penicillin 10.000 U/mL and streptomycin 10.000 mg/mL), in 75 cm^2^ culture flasks (Falcon, Mountain View, CA) until they had formed a confluent monolayer.

### 2.4. Transfection

The human RLN2 siRNA 1–3 and scrambled control siRNA (control siRNA) transfections (MG-63/siRNA) were performed using Lipofectamine 2000 (Invitrogen, Carlsbad, CA, USA) in Opti-MEM (Invitrogen) according to the manufacturer's protocol with a final siRNA concentration of 100 nM. The transfection reagent was removed after 12 h and the cells were harvested after 48 h. AKT/NF-*κ*B signal and MMPs, VEGF, and Bcl-2 expression were detected as below. To study whether RLN2-induced MMPs, VEGF, and Bcl-2 expression were via AKT/NF-*κ*B signal, the MG-63/siRNA cells were transfected with myr-Akt as described above or treated with 20 ng/mL of TNF-*α* for 6 hs to activate AKT or NF-*κ*B.

To further investigate the correlation between RLN2 and AKT/NF-*κ*B signal, U-2OS cells were treated with 100 nM recombinant relaxin for 24 hs or treated with NF-*κ*B inhibitor BAY 11-7082 (10 *μ*M) or transfected with I*κ*B*α*M (100 nM) or LY294002 (50 *μ*M) for 24 h (3 h for LY294002) before B-29/A-24 treatment.

### 2.5. Matrigel Invasion Assay

The invasiveness of OS cells was tested after transfection and treatment at different time points described. The cells (1 × 10^6^/mL) were added to the upper wells coated with Matrigel (1 mg/mL, Collaborative Research, Inc., Boston, MA) with serum-free medium containing 25 *μ*g/mL fibronectin as a chemoattractive agent in the lower wells. After 24 hs of incubation period, cells that migrated through the filters into the lower chamber were counted by the number of cells on the lower side of the membrane in five random fields after staining with Hema-3 kit.

### 2.6. In Vitro Angiogenesis Assay [3]

The conditioned medium of OS cells at different time points was filtered off for future research. HMEC-1 cells (human umbilical vein endothelial cell) (4 × 10^4^) were seeded onto eight-well chamber slides and the aforementioned conditioned medium was added. Cells were cultured for 72 hs until capillary network formation was observed. The number of branch points and total number of branches per point were counted after H&E staining to quantify the degree of angiogenesis.

### 2.7. Cell Growth Inhibition by 3-(4,5-Dimethylthiazol-2-yl)-2,5-diphenyltetrazolium Bromide Assay (MTT)

To detect the effect of RLN2 inhibition on* cell growth*, MG-63 cells (10^4^ cells/well) were seeded on 96-well plates in 100 *μ*L of complete culture medium, allowed to attach for 24 hs, and then transfected with RLN2 siRNA [1–3] or control siRNA mixtures for 0–5 days as per the manufacturer's instructions.

To detect the effect of RLN2 overexpression on* cell growth*, U-2OS cells (10^4^ cells/well) were seeded on 96-well plates in 100 *μ*L of complete culture medium, allowed to attach for 24 hs, and then treated with 100 nM recombinant relaxin for 0–5 days.

At different time points, 200 *μ*L sterile MTT dye (5 mg/mL, Sigma, USA) was added to each well. After 4 hs of incubation at 37°C in 5% CO_2_, MTT medium mixture was removed and 200 *μ*L of dimethyl sulfoxide (DMSO) was added to each well and incubated further for 2 hs. Upon termination, the supernatant was aspirated and the MTT formazan formed by metabolically viable cells was dissolved in 100 *μ*L of isopropanol. The plates were mixed for 30 minutes on a gyratory shaker, and absorbance was measured at 490 nm using a multiwell spectrophotometer (Thermo Electron, Andover, USA). All experiments were carried out in triplicate.

### 2.8. Apoptosis Assay

Cells in each well at different time points were harvested and cell apoptosis was detected by Annexin V-FITC/PI staining method. The experiments were done in triplicate for each sample, and analyses were performed using a FACScan flow cytometer (Becton-Dickinson) in accordance with the manufacturer's guidelines.

### 2.9. Chemosensitivity Assay

MG-63 and U-2OS cells (1 × 10^4^ cells/well) at different time points were cisplatin (10 *μ*g/mL) for 48 hs. Cell chemosensitivity to cisplatin was evaluated by apoptotic analysis as above.

### 2.10. Western Blotting

Total protein from the OS tissues or cells at different time points was extracted using RIPA lysis buffer containing 60 *μ*g/mL PMSF. Protein concentrations were determined by BCA protein assay kit (Boster, China). The protein samples were denatured at 100°C for 10 min and then preserved at −20°C for later use. The protein samples were separated by 8% SDS-polyacrylamide gels and transblotted onto nitrocellulose blotting membrane (0.22 *μ*m). Membranes were blocked with 5% skim milk for 1 h at room temperature and probed with primary antibodies (rabbit anti-RLN2, anti-LGR7 rabbit, anti-NF-*κ*B (p65), anti-p-AKT (Ser473), and anti-pERK1/2, 1 : 1000, goat anti-MMP-2/9, and anti-bcl-2, 1 : 1000; mouse anti-*β*-actin, 1 : 2000) overnight at 4°C. After incubation with the appropriate anti-rabbit, anti-goat, or anti-mouse horseradish peroxidase-conjugated secondary antibody (1 : 5000, Boster, China) for 1.5 hs at room temperature, immunoreactive bands were visualized by the chemiluminescence dissolvent (Thermo, USA) and exposed to the X-ray film (Kodak, USA). The determination of grayscale value was processed by Image J. All experiments were repeated by six times over multiple days.

### 2.11. Gelatinolytic Zymography

Analysis of MMP-9 activity was carried out in 7.5% (w/v) SDS-PAGE containing 0.1% gelatin (w/v). Equal amounts of culture media (20 *μ*L) were applied to the gel in Laemmli sample buffer lacking *β*-mercaptoethanol. Samples were preincubated for 60 min with 0.5 mm aminophenylmercuric acid (APMA, Sigma) which activates the proform to the activated form. Gelatinolytic activities of active MMP-9 were detected as transparent bands on the background of Coomassie Blue-stained gelatin. The NIH Image 1.44 *β*11 software was used for the analysis of the bands, after acquisition in an Appligene densitometer (Oncor).

### 2.12. Electrophoretic Mobility Shift Assay (EMSA)

Proteins were extracted by freeze-thaw lysis using buffer C for electrophoretic mobility shift assays. EMSA experiments were done as per the manufacturer's instructions. Briefly, protein extracts (6 *μ*g) were incubated for 30 min at room temperature with radiolabeled DNA probes containing a consensus kB site, separated on a native polyacrylamide gel, and visualised by autoradiography. Retinoblastoma protein level served as nuclear protein loading control.

### 2.13. Statistical Analysis

All experiments were conducted in triplicate and carried out on three or more separate occasions. Data presented are means of the three or more independent experiments ± SE. Statistically significant differences were determined by Student's *t*-test and were defined as ^*∗*^ *P* < 0.05. All analyses were performed with SPSS version 13.0 software.

## 3. Results

### 3.1. Positive Correlation between RLN2 and p-AKT (Ser473) Protein Expression

**Figure 1:**
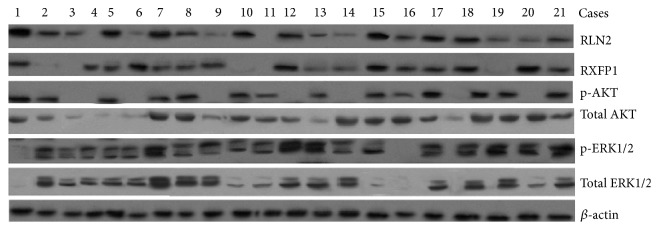
Western blot assay for RLN2, RXFP1, AKT, ERK1/2, p-Akt, and p-ERK1/2 in OS tissues with pulmonary metastatic disease.

In order to investigate the correlation between RLN2 and RXFP1 (LGR7), RLN2 and p-AKT, and RLN2 and p-ERK1/2 in OS tissues which exist in pulmonary metastatic disease, the RLN2, RXFP1, AKT, ERK1/2, p-AKT, and p-ERK1/2 protein in 21 samples from patients with pulmonary metastatic disease were detected by western blot assay (Figure 1). There was a significant positive correlation between RLN2 and RXFP1 expression (*R* = 0.794, *P* = 0.025) and RLN2 and p-AKT expression (*R* = 0.835, *P* = 0.016). No correlation was found between RLN2 and p-ERK1/2 expression (*R* = 0.359, *P* = 0.083). These data suggest that a possible connection between RLN2 expression and the phosphorylation of AKT exists in OS.

### 3.2. Specific siRNA Inhibited RLN2 Expression in MG-63 Cells

**Figure 2:**
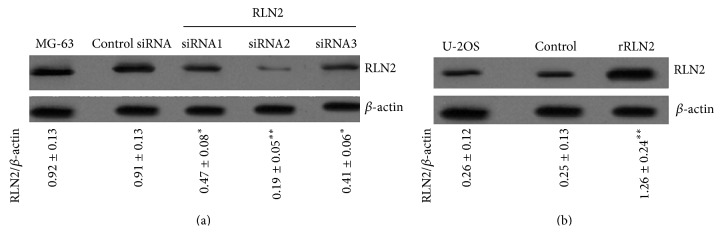
Expression of RLN2 in OS cells following different treatment. (a) The expression of RLN2 protein was measured by western blot in MG-63 cells with specific siRNA transfection. The result showed that RLN2 was significantly blocked in positive groups compared with control group. (b) U-2OS cells were treated with 100 nM recombinant relaxin for 24 hs. The expression of RLN2 protein was measured by western blot in MG-63 cells. The result showed that RLN2 was significantly increased in positive groups compared with control group. ^*∗*^ *P* < 0.05; ^*∗∗*^ *P* < 0.01, versus control.

In order to investigate effect of RLN2 inhibition in the subsequent experiments, the RLN2 siRNA1, RLN2 siRNA2, and RLN2 siRNA3 were used to inhibit RLN2 expression in MG-63 cells. The result of western blot assays shows that the RLN2 protein was significantly lower in cells transfected with RLN2 siRNA than in those transfected with control siRNA (Figure 2(a), ^*∗*^ *P* < 0.05, ^*∗∗*^ *P* < 0.01). RLN2 siRNA2 has the highest effect on targeting RLN2, so RLN2 siRNA2 was used for further study.

To study the effect of RLN2 overexpression on OS cells, U-2OS cells were treated with 100 *μ*M recombinant human RLN2 (B-29/A-24) for 24 hs. The result of western blot assays shows that the RLN2 protein was significantly increased in the U-2OS cells more than in those cells transfected with control siRNA (Figure 2(b), ^*∗∗*^ *P* < 0.01).

### 3.3. Silencing RLN2 Decreased AKT/NF-*κ*B Signaling Pathway in MG-63 Cells

**Figure 3:**
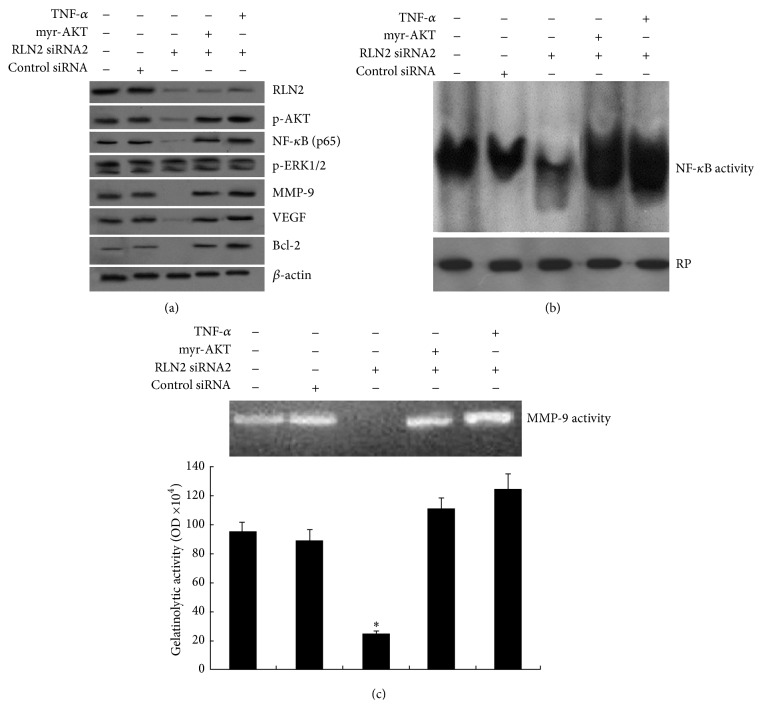
Effect of RLN2 inhibition decreased AKT/NF-*κ*B signaling pathway in MG-63 cells. MG-63 cells were transfected with RLN2 siRNA2 for 48 hs and then treated with myr-AKT or TNF-*α* at different time points. (a) The protein of p-AKT (Ser473), p-ERK1/2, NF-*κ*B (p65), MMP-9, VEGF, and bcl-2 was measured using western blot analysis. (b) Nuclear extracts were prepared and subjected to analysis for NF-*κ*B DNA-binding activity as measured by EMSA. Retinoblastoma protein level served as nuclear protein loading control. (c) Zymographic analysis of MMP-9 gelatinolytic activity, which was quantified by densitometry and graphed in OD units (mean ± SEM) (^*∗*^ *P* < 0.05).

In order to investigate the effect of RLN2 inhibition on the AKT/NF-*κ*B signaling pathway in OS cells, the MG-63 cells were transfected with RLN2 siRNA2 for 48 hs. The protein of p-AKT (Ser473), p-ERK1/2, NF-*κ*B (p65), MMP-9, VEGF, and bcl-2 was measured using western blot analysis. NF-*κ*B activity was detected by EMSA; MMP-9 activity was detected by Gelatinolytic Zymography. Results revealed that p-AKT (0.83 ± 0.14 versus 0.16 ± 0.07), p65 (0.52 ± 0.17 versus 0.19 ± 0.12), MMP-9 (0.46 ± 0.14 versus 0.09 ± 0.00), VEGF (0.39 ± 0.02 versus 0.12 ± 0.02), and bcl-2 (0.48 ± 0.1 versus 0.14 ± 0.03) expression (Figure 3(a)) and NF-*κ*B (Figure 3(b)) and MMP-9 activity (Figure 3(c)) were inhibited compared to the control siRNA (*P* < 0.05, resp.). No significant change of p-ERK1/2 activity was found (Figure 3(a)).

When RLN2 siRNA2 transfected MG-63 cells (MG-63/RLN2 siRNA2) were transfected with myr-AKT (10 *μ*M) for 24 hs, p-AKT (0.16 ± 0.07 versus 0.84 ± 0.12), NF-*κ*B (0.19 ± 0.12 versus 0.74 ± 0.13), MMP-9 (0.09 ± 0.00 versus 0.54 ± 0.11), VEGF (0.09 ± 0.00 versus 0.48 ± 0.09), and bcl-2 (0.12 ± 0.00 versus 0.53 ± 0.13) expression was significantly increased compared to the MG-63/RLN2 siRNA2 groups (*P* < 0.05, resp.) using western blot analysis (Figure 3(a)). NF-*κ*B (Figure 3(b)) and MMP-9 activity (Figure 3(c)) was also increased compared to the cells treated with RLN2 siRNA2 alone.

When MG-63/RLN2 siRNA2 cells were treated with 20 ng/mL of TNF-*α* for 6 hs, NF-*κ*B (p65) (0.19 ± 0.12 versus 0.92 ± 0.23), MMP-9 (0.09 ± 0.00 versus 0.64 ± 0.17), VEGF (0.12 ± 0.02 versus 0.53 ± 0.13), and bcl-2 (0.12 ± 0.00 versus 0.57 ± 0.14) expression was significantly increased compared to the MG-63/RLN2 siRNA2 groups (*P* < 0.05, resp.) (Figure 3(a)). NF-*κ*B (Figure 3(b)) and MMP-9 activity (Figure 3(c)) was also increased compared to the RLN2 siRNA2 alone. No significant change of p-AKT activity was found (Figure 3(a)). Furthermore, TNF-*α* treatment did not induce p-AKT activity in the MG-63 cells (data not shown).

### 3.4. RLN2 Overexpression Increased AKT/NF-*κ*B Signaling Pathway in U-2OS Cells

**Figure 4:**
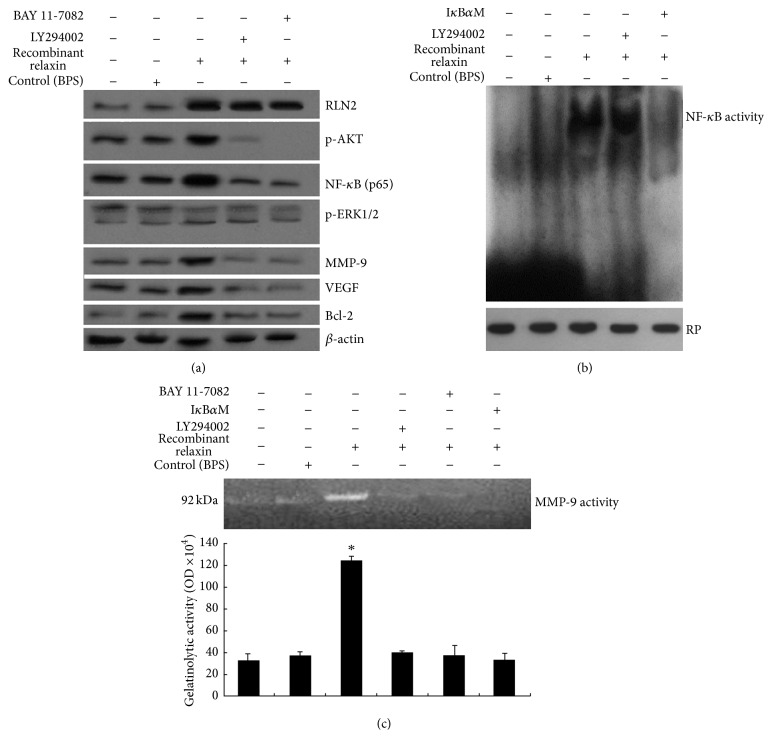
Effect of RLN2 overexpression increased AKT/NF-*κ*B signaling pathway in U-2OS cells. (a) U-2OS cells were treated with different agents at different time points. The protein of p-AKT (Ser473), p-ERK1/2, NF-*κ*B (p65), MMP-9, VEGF, and bcl-2 was measured using western blot analysis. (b) U-2OS cells were treated with different agents at different time points. Nuclear proteins were extracted by freeze-thaw lysis using buffer C in U-2OS cells for electrophoretic mobility shift assays (EMSA). Retinoblastoma protein level served as nuclear protein loading control. (c) U-2OS cells were treated with different agents at different time points. Zymographic analysis of MMP-9 gelatinolytic activity, which was quantified by densitometry and graphed in OD units (mean ± SEM) (^*∗*^ *P* < 0.05).

U-2OS cells were treated with 100 nM recombinant relaxin for 24 hs. The protein of p-AKT (Ser473), NF-*κ*B (p65), MMP-9, VEGF, and bcl-2 expression measured using western blot analysis was increased compared to the cells treated with control PBS (Figure 4(a)). NF-*κ*B activity (Figure 4(b)) and MMP-9 activity (Figure 4(c)) were also increased by EMSA and Gelatinolytic Zymography. No significant change of p-ERK1/2 activity was found (Figure 4(a)).

When U-2OS cells were treated with 50 *μ*M LY294002 for 3 hs and then treated with 100 nM recombinant human RLN2 (B-29/A-24) for 24 hs, phosphorylation of AKT (p-AKT) that B-29/A-24 induced was completely inhibited (Figure 4(a)). Furthermore, NF-*κ*B (p65), MMP-9, VEGF, and bcl-2 expression was also inhibited (Figure 4(a)). NF-*κ*B activity (Figure 4(b)) and MMP-9 activity (Figure 4(c)) were also decreased compared to the U-2OS cells treated with B-29/A-24 alone.

When U-2OS cells were treated with NF-*κ*B inhibitor BAY 11-7082 (10 *μ*M) for 24 hs and then treated with 100 nM recombinant relaxin for 24 hs, NF-*κ*B (p65), MMP-9, VEGF, and bcl-2 expression was inhibited compared to the cells treated with B-29/A-24 alone (Figure 4(a)). MMP-9 activity (Figure 4(c)) was also inhibited.

When U-2OS cells were transfected with I*κ*B*α*M (100 nM) for 24 hs and then treated with 100 nM recombinant relaxin for 24 hs, MMP-9, VEGF, and bcl-2 expression was inhibited (data not shown). NF-*κ*B (Figure 4(b)) and MMP-9 activity (Figure 4(c)) was also inhibited.

### 3.5. Silencing RLN2 Inhibits MG-63 Cell Growth

**Figure 5:**
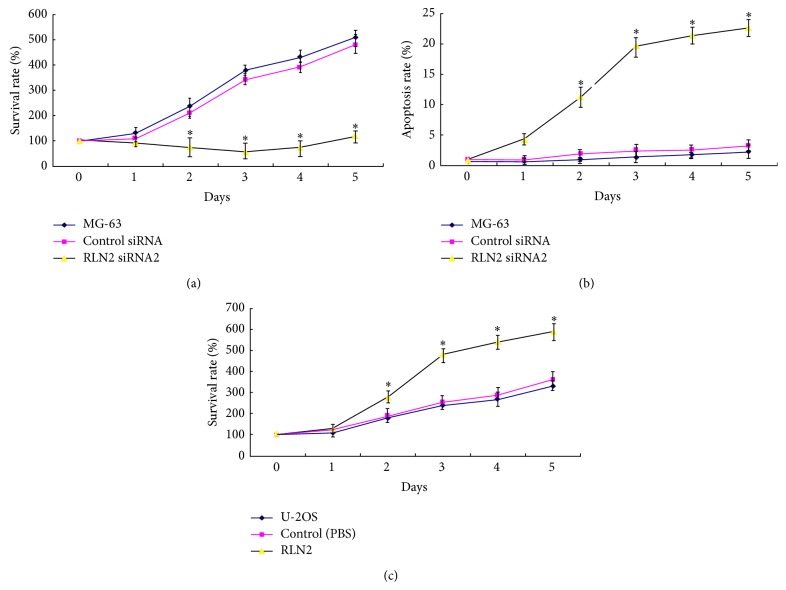
Effect of RLN2 on cell growth and apoptosis. (a) RLN2 siRNA2 was transfected into the MG-63 cell for 1–5 days; cell survival rate was performed with MTT assay. (b) Cell apoptosis was detected by Annexin V-FITC/PI staining method. (c) U-2OS cells were treated with recombinant relaxin for 5 days, and then we performed determination of cell survival rate with MTT assay. ^*∗*^ *P* < 0.05, versus control.

To determine whether RLN2 siRNA2 had an inhibitory effect on MG-63 cell growth, we first performed determination of cell survival rate with MTT assay.  Figure 5(a) showed that the growth curves for RLN2 siRNA2 silencing cells were significantly lower than those for control cells in 5 days of incubation. Cells at different time points were harvested and cell apoptosis was detected by Annexin V-FITC/PI staining method.  Figure 5(b) showed that the apoptotic rate was significantly higher in RLN2 siRNA2 silencing cells than those for control cells in 5 days of incubation. This result indicated that siRNA inhibited OS cell growth and promoted apoptosis.

### 3.6. RLN2 Overexpression Promotes U-2OS Cell Growth

To determine whether RLN2 had a promotional effect on* U-2OS* cell growth,* U-2OS cells* were treated with recombinant relaxin and then we performed determination of cell survival rate with MTT assay.  Figure 5(c) showed that the growth curves for RLN2 treated cells were significantly higher than those for control cells in 5 days of incubation. Cells at different time points were harvested and cell apoptosis was detected by Annexin V-FITC/PI staining method. No effect of RLN2 treatment alone was found on cell apoptosis (data not shown).

### 3.7. Silencing RLN2 Increases Sensitivity of MG-63 Cells to Cisplatin

**Figure 6:**
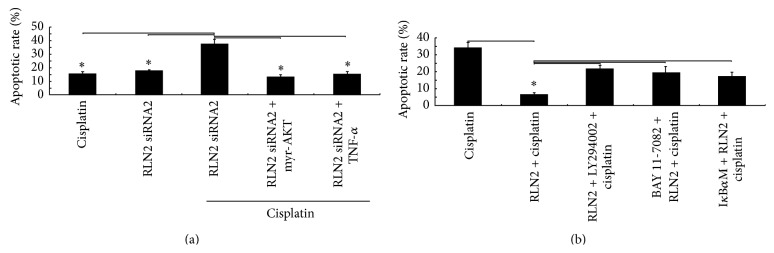
RLN2 regulates sensitivity of OS cells to cisplatin. (a) MG-63 cells were transfected with RLN2 siRNA2 and then treated with myr-AKT (10 *μ*M) for 24 hs or treated with 20 ng/mL of TNF-*α* for 6 hs, then following 10 *μ*g/mL cisplatin treatment for 48 hs. (b) U-2OS cells were treated with NF-*κ*B inhibitor BAY 11-7082 (10 *μ*M) for 24 hs, or 50 *μ*M LY294002 for 3 hs, or I*κ*B*α*M (100 nM) for 24 hs; then, the cells were treated with recombinant relaxin for 24 hs, after which the cells were treated with 10 *μ*g/mL cisplatin for 48 hs. Cell apoptosis was detected by Annexin V-FITC/PI staining method. The experiments were done in triplicate for each sample, and analyses were performed using a FACScan flow cytometer (^*∗*^ *P* < 0.05).

Only low levels (<20%) of apoptosis were detected in MG-63 cells following 10 *μ*g/mL cisplatin treatment (Figure 6(a)). This might be due to the endogenous RLN2 and/or simultaneous induction of the antiapoptotic RLN2 targets by cisplatin. Indeed, RLN2* inhibition* by siRNA led to a significant increase in cisplatin-induced apoptosis (Figure 6(a)), suggesting that combining* RLN2* inhibition with cisplatin increased the incidence of apoptosis.

### 3.8. RLN2 Overexpression Decreases Sensitivity of U-2OS Cells to Cisplatin

34% of apoptotic rate was detected in U-2OS cells following 10 *μ*g/mL cisplatin treatment (Figure 6(b)). However, when the U-2OS cells were treated with recombinant relaxin, then following 10 *μ*g/mL cisplatin treatment, only 4.8% of apoptotic rate was detected in the U-2OS cells, suggesting that* RLN2* inhibited cisplatin-induced apoptosis in U-2OS cells.

### 3.9. RLN2 Regulates Sensitivity of OS Cells to Cisplatin by AKT/NF-*κ*B Signaling Pathway

When RLN2 siRNA2 transfected MG-63 cells (MG-63/RLN2 siRNA2) were transfected with myr-AKT (10 *μ*M) for 24 hs or treated with 20 ng/mL of TNF-*α* for 6 hs, then following 10 *μ*g/mL cisplatin treatment for 48 hs, RLN2 siRNA2 led to a significant decrease in cisplatin-induced apoptosis (Figure 6(a)), respectively.

When U-2OS cells were treated with NF-*κ*B inhibitor BAY 11-7082 (10 *μ*M) for 24 hs, or 50 *μ*M LY294002 for 3 hs, or I*κ*B*α*M (100 nM) for 24 hs, then the cells were treated with recombinant relaxin for 24 hs, after which the cells were treated with 10 *μ*g/mL cisplatin for 48 hs. The results showed that RLN2 overexpression led to a significant increase in cisplatin-induced apoptosis (Figure 6(b)), respectively.

### 3.10. Effect of RLN2 on Invasion and Angiogenesis via AKT/NF-*κ*B Signaling Pathway

**Figure 7:**
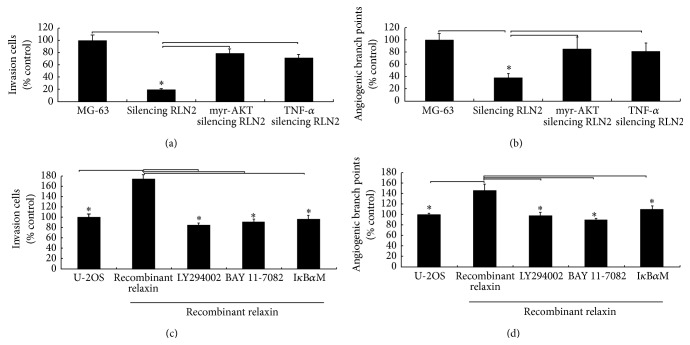
Effect of RLN2 on invasion and angiogenesis in OS cells. MG-63 cells were transfected with RLN2 siRNA2 for 48 hs and then transfected with myr-AKT (10 *μ*M) for 24 hs or treated with 20 ng/mL of TNF-*α* for 6 hs; the invasive ability of MG-63 cells was detected by Matrigel invasion assay (a). Quantitative analysis of the tube formation by HUVECs induced by conditioned medium (b). The reduced invasion and tube formation induced by the conditioned medium of RLN2-siRNA2 transfected MG-63 cells were rescued by adding myr-AKT or TNF-*α*. U-2OS cells were treated with NF-*κ*B inhibitor BAY 11-7082 (10 *μ*M) for 24 hs, or 50 *μ*M LY294002 for 3 hs, or I*κ*B*α*M (100 nM) for 24 hs; then, the cells were treated with recombinant relaxin for 24 hs; the invasive ability of U-2OS cells was detected by Matrigel invasion assay (c). Quantitative analysis of the tube formation by HUVECs induced by conditioned medium (d). The increased invasion and tube formation induced by the conditioned medium of RLN2 treated U-2OS cells were rescued by adding BAY 11-7082 or LY294002 or I*κ*B*α*M. ^*∗*^ *P* < 0.05.

The result (Figure 7(a)) from Matrigel invasion assay indicates that RLN2 silence significantly inhibited the invasion of MG-63 cells by 84.6%, as compared with mock-transfected and control cells. Furthermore, the results (Figure 7(b)) showed that HMECs treated with conditioned media from mock and MG-63 cells were able to form capillary-like structures. It shows that HMECs treated with conditioned media from RLN2 siRNA-transfected MG-63 cells showed fewer capillary-like networks, as compared with the controls. When RLN2 siRNA2 transfected MG-63 cells (MG-63/RLN2 siRNA2) were transfected with myr-AKT (10 *μ*M) for 24 hs or treated with 20 ng/mL of TNF-*α* for 6 hs, the invasive ability of MG-63 cells was significantly increased as compared with the RLN2 inhibition alone (Figure 7(a)). More capillary-like networks were shown, as compared with RLN2 inhibition alone (Figure 7(b)).

Furthermore, we found from Matrigel invasion assay that U-2OS cells treated with recombinant relaxin for 24 hs significantly promoted the invasion of U-2OS cells by 62.4%, as compared with control cells (Figure 7(c)). Furthermore, the results (Figure 7(d)) showed that HMECs treated with conditioned media from recombinant relaxin treated U-2OS cells showed more capillary-like networks. When U-2OS cells were treated with NF-*κ*B inhibitor BAY 11-7082 (10 *μ*M) for 24 hs, or 50 *μ*M LY294002 for 3 hs, or I*κ*B*α*M (100 nM) for 24 hs, then the cells were treated with recombinant relaxin for 24 hs; the invasive ability of U-2OS cells was significantly decreased as compared with recombinant relaxin treatment alone (Figure 7(c)). Fewer capillary-like networks were also shown, as compared with recombinant relaxin treatment alone (Figure 7(d)).

## 4. Discussion

Despite having two peptide-coding genes, relaxin gene 1 (RLN1) and RLN2, the major stored and circulatory form of relaxin in humans is relaxin-2. Relaxin-1 is a pseudogene, which does not translate into a functional peptide in rodents, humans, and other nonhuman species. Relaxin-2 is produced in the prostate by males [30] and corpus lutea in females [31]. RXFP1 (LGR7) is a cognate receptor for RLN2; RXFP2 (LGR8) is a cognate receptor for INSL3.

Two receptors responding to relaxin, LGR7 and LGR8, were cloned and described in 2002 [32, 33]. In transfected cell systems, these receptors are shown to respond to relaxin by causing an elevation of intracellular cAMP, presumably through a G-protein-dependent activation of adenylate cyclase [32]. In human primary endometrial stromal cells, a similar elevation in cAMP is detected upon relaxin stimulation, but here inhibition of intracellular phosphodiesterases also appears to be playing an important role [34, 35]. Interestingly, pharmacological studies show that for the human LGR8 both the relaxin and the peptide INSL3 identified over a decade ago (also called relaxin-like factor, RLF) can act as effective ligands, whereas LGR7 only responds to relaxin [33].

While well known for RLN2's reproductive and antifibrotic roles, relaxin has been associated with neovascularization of the endometrial lining of the uterus, potentially via specific induction of vascular endothelial growth factor (VEGF) [36]. Silvertown et al. have found prostate cancer xenografts overexpressing RLN2 exhibited greater tumor volumes and angiogenesis. An advanced angiogenic phenotype and increased VEGF transcript were observed in RLN2 overexpressed tumors [37]. Our previous study also found RLN2 was related with OS tumor growth, neovascularization, metastasis, and oncogenic progression [3], but the underlying molecular mechanisms of this action are largely unknown.

AKT is a “master regulator” that when activated by phosphorylation modifies at least ten major regulatory proteins. It is important in initiation of many pathways in both normal and tumor cells. These play a central role in a variety of oncogenic processes including cell growth, proliferation, apoptotic cell death, motility, epithelial-mesenchymal transition (EMT), angiogenesis, and metastasis [38–41].

Membrane bound AKT is activated by two phosphorylations, by PDK1 at Thr-308 on the activation loop, and by mTORC2 at Ser-473 on the hydrophobic motif. These are independent in sequence, and both are necessary for full activation of AKT. It has been recently found that RLN2 could increase phosphorylation of AKT [42, 43], which suggested that a possible connection between RLN2 and p-AKT expression exists.

In the next set of experiments, we extended our analyses of the involved molecules and pathways by applying the gene targeting technology. In these experiments, we have first detected the RLN2 and p-AKT protein expression in 21 samples from patients with pulmonary metastatic disease, only to find that RLN2 and p-AKT protein was significantly increased in OS tissues which exist in pulmonary metastatic disease (Figure 1), and a significant positive correction between RLN2 and p-AKT expression was found (Figure 1).

To further investigate the pathway of RLN2-mediated effects, we analyzed the p-AKT and NF-*κ*B family known to be critically involved in cell invasion and angiogenesis formation. In our present study, we observed that RLN2 overexpression increased p-AKT, which is a kinase involved in diverse pathways activating NF-*κ*B (Figure 3). Moreover, p-AKT inhibitor LY294002 inhibited the NF-*κ*B expression and activity (Figure 3). Otherwise, we found that RLN2 inhibition decreased p-AKT expression and NF-*κ*B activity. However, when the cells were treated with p-AKT activator myr-AKT, NF-*κ*B activity and expression were significantly increased. Moreover, NF-*κ*B inhibitor BAY 11-7082, NF-*κ*B activator TNF-*α*, and super repressor of NF-*κ*B activity I*κ*B*α*M did not increase or decrease p-AKT activity, confirming the involvement of RLN2/p-Akt/NF-*κ*B pathway.

We used RLN2 siRNA to inhibit the RLN2 expression and demonstrated two major consequences in MG-63 cells. First, in the absence of RLN2 activity, leading to decreased cell proliferation and angiogenesis formation and invasion. Second, inhibition of RLN2 function leads to decreased activation of the AKT/NF-*κ*B signaling pathway, resulting in reduced cell survival of MG-63 cells. Otherwise, we used recombinant human RLN2 (B-29/A-24) treatment to increase the RLN2 expression and also demonstrated two major consequences in U-2OS cells. First, increase of RLN2 activity leads to increased cell proliferation, angiogenesis formation, and invasion. Second elevated RLN2 function leads to increased activation of the AKT/NF-*κ*B signaling pathway, resulting in elevated cell survival of U-2OS cells. Our data showed for the first time that RLN2 changed the invasive potential of OS cell lines through AKT/NF-*κ*B signaling pathway.

Expression of VEGF by tumor cells directly correlates with angiogenesis, which contributes to the progressive growth of human OS [44–46]. Matrix metalloproteinase-9 (MMP-9) plays an important role in the progression of OS by increasing tumor growth, migration, invasion, and metastasis and is associated with poor disease prognosis [47–49]. The regulation of MMP-9 expression during tumor progression may involve diverse mechanisms. Many studies have found that production of MMP-9 and VEGF was via the AKT/NF-*κ*B signaling pathway [50–57].

Although it is well established that the RLN2 is essential for AKT/NF-*κ*B activity in our study, it is not known whether RLN2 regulates AKT/NF-*κ*B dependent-VEGF and MMP-9 expression. The results showed that LY294002, BAY 11-7082, and I*κ*B*α*M treatment suppressed RLN2-induced production of VEGF and MMP-9. TNF-*α* and myr-AKT treatment rescued the production of VEGF and MMP-9 after RLN2 was inhibited by siRNA transfection. We therefore concluded that overexpression of RLN2 in OS cells leads to augmented production of MMP-9 and VEGF via activation of AKT/NF-*κ*B signaling, and vice versa. We also observed that RLN2 regulated angiogenesis formation and invasion by regulating RLN2/AKT/NF-*κ*B dependent-VEGF and MMP-9 expression. However, in vivo prostate xenograft tumor RLN2 inhibited pro-MMP-9 expression [42], which was opposite to our study. This may be the cause of tissue specificity.

The PI3K/Akt signaling pathway is constitutively activated in some cancers; when activated, it inhibits chemotherapy-mediated apoptosis; when inhibited, it promotes chemotherapy-mediated apoptosis [24–26, 58, 59]. In colorectal carcinomas and breast cancer, inhibition of AKT sensitizes cells to the apoptotic effect of chemotherapy by transcriptional inhibition of NF-*κ*B signaling [58, 59]. In pancreatic cancer, inhibition of either phosphatidylinositol-3 kinase or AKT led to a decreased protein level of the antiapoptotic gene BCL-2 and an increased protein level of the proapoptotic gene BAX. Furthermore, inhibition of AKT decreased the function of NF-*κ*B, which is capable of transcriptional regulation of the BCL-2 gene [25]. Zhang et al. have found AKT2 inhibition sensitized gemcitabine-induced apoptosis via PUMA upregulation in MIAPaCa-2 cells in vitro, and via NF-*κ*B activity inhibition in L3.6pl cells in vitro. In PANC-1 and MIAPaCa-2 cells in vivo, AKT2 inhibition sensitized gemcitabine-induced apoptosis and growth inhibition via both PUMA upregulation and NF-*κ*B inhibition [24]. In our present study, we observed that RLN2 overexpression decreased the chemosensitivity to cisplatin in OS cells via induction of AKT/NF-*κ*B/bcl-2 signaling. Moreover, RLN2 inhibition increased the chemosensitivity to cisplatin in OS cells via inhibition of AKT/NF-*κ*B/bcl-2 signaling. The antiapoptotic effect of RLN2 activation in OS cells may involve transcriptional induction of BCL-2 proteins that confer resistance to apoptosis via AKT/NF-*κ*B signaling; alteration of this balance allows sensitization to the apoptotic effect of chemotherapy.

Extracellular signal-regulated kinase (ERK) is a member of the mitogen-activated protein kinase (MAPK) family, which regulates essential cellular functions like proliferation, differentiation, cell survival, and cell death. ERK1/2 signaling mediates chemoresistance in the cells and could be important therapeutic targets for overcoming chemoresistance in OS [22]. In our study, we found that no matter RLN2 overexpression or RLN2 silencing, ERK1/2 did not change. We therefore suggested that ERK1/2 was not regulated by RLN2 under our experimental conditions in OS cells.

In conclusion, RLN2 overexpression was related to metastasis in OS cases. We also found the ability of RLN2 to stimulate effect of cancer cell invasion, proliferation, and angiogenesis formation and decrease sensitivity of OS cells to chemotherapy; the sharp reduction of cancer cell invasiveness, angiogenesis formation, and the increase of sensitivity of OS cells to chemotherapy after RLN2 gene were knockdown. Our results indicated that RLN2 confers to migratory and invasive ability, angiogenesis, and chemoresistance to cisplatin via modulating the AKT/NF-*κ*B signaling pathway in vitro. The RLN2 blocker may be a new therapeutic strategy in OS management.
